# Prognostic Impact of Polypharmacy following Trans-Catheter Aortic Valve Replacement

**DOI:** 10.3390/jcm12072598

**Published:** 2023-03-30

**Authors:** Teruhiko Imamura, Nikhil Narang, Ryuichi Ushijima, Mitsuo Sobajima, Nobuyuki Fukuda, Hiroshi Ueno, Koichiro Kinugawa

**Affiliations:** 1 The Second Department of Internal Medicine, University of Toyama, 2630 Sugitani, Toyama 930-0194, Japan; 2 Advocate Christ Medical Center, Oak Lawn, IL 60453, USA

**Keywords:** heart failure, hemodynamics, medication, aortic valve disease

## Abstract

Background: Polypharmacy in elderly patients with various comorbidities is associated with mortality and morbidity. However, the prognostic impact of polypharmacy in patients with severe aortic stenosis receiving trans-catheter aortic valve replacement remains unknown. Methods: Patients with severe aortic stenosis who received trans-catheter aortic valve replacement between 2015 and 2022 and were followed up at our institute following index discharge were included in this retrospective study. The impact of polypharmacy, which was defined as medication numbers ≥10 at index discharge, upon 2-year all-cause death was investigated. Results: A total of 345 patients (median age 85 [83, 89] years old, 99 (29%) men) were included. Median medication number was 9 (7, 10) at the index discharge and 88 (26%) were classified as receiving polypharmacy. Frailty index, including mini-mental state examination and CSHA score, were not significantly different between those with and without polypharmacy (*p* > 0.05 for both). Polypharmacy was associated with higher 2-year cumulative mortality with an adjusted hazard ratio of 21.4 (95% confidence interval, 6.06–74.8, *p* < 0.001). As a sub-analysis, the number of cardiovascular medications was not associated with 2-year mortality (hazard ratio 1.12, 95% confidence interval 0.86–1.48, *p* = 0.46), whereas a higher number of non-cardiovascular medications was associated with an incremental increase in 2-year mortality with a hazard ratio of 1.39 (95% confidence interval, 1.15–1.63, *p* < 0.001). Conclusions: In elderly patients with severe aortic stenosis, polypharmacy was associated with worse short-term survival following trans-catheter aortic valve replacement. Prognostic implication of aggressive intervention to decrease the amount of medication among those receiving TAVR requires further prospective studies.

## 1. Background

Trans-catheter aortic valve replacement (TAVR) has been introduced as less invasive trans-catheter intervention to treat severe aortic stenosis, initially for those with a high-risk for surgical valve replacement. Clinical outcome following TAVR, including safety of the procedure and reduction of procedure-related complication, has improved considerably with the introduction of a new generation of device design, establishment of dedicated imaging analyses for pre-procedural planning, including valve and arterial access selection, optimal patient selection, minimization of procedure, including single arterial access and conscious sedation, transition from dual to single antiplatelet therapy, and several technical enhancements [1,2]. Thus, indication of TAVR has expanded to younger and lower-risk patients, leading to an expansion of current guideline recommendations for TAVR. Nevertheless, some patients have yet to experience a higher rate of morbidity and mortality, due to a high risk baseline clinical risk profile [3]. Of note, many patients with severe aortic stenosis are both elderly and have a high burden of other comorbid conditions, all of which may contribute to early post procedure mortality, despite TAVR [4]. The existence of multi-comorbidities, which is represented as frailty or sarcopenia, is receiving great concern as one of the critical risk factors for mortality and morbidity following TAVR.

Polypharmacy is one of the major growing issues in the clinical management of elderly patients [5]. Polypharmacy represents the existence of multi-comorbid conditions for which the aggregate daily pill burden for many patients can be considerable [6]. On top of the challenges of managing complex medication regimens, the pharmacologic interaction of multiple drugs in patients with several comorbid conditions may dramatically affect health status. Previous studies have demonstrated a strong association between polypharmacy and poor clinical outcomes in geriatric patients, with or without heart failure [7,8,9,10].

However, the clinical implications of polypharmacy for those receiving TAVR has not been well studied. Most candidates for TAVR have multiple comorbidities and probably receive polypharmacy. Their comorbidity, instead of valvular disease itself, seems to have considerable prognostic impact. Altogether, the existence of polypharmacy might be a key for risk stratifying TAVR candidates. Such knowledge should be of great importance for elderly patients in indicating TAVR procedure and considering post-TAVR management. Thus, this study aimed to investigate the prognostic impact of polypharmacy following TAVR.

## 2. Methods

### 2.1. Patient Selection

Patients with severe aortic stenosis who underwent TAVR at our institute during index hospitalization between 2015 May and 2022 June were prospectively registered in the institutional registry database and considered for inclusion in this retrospective study. Patients who died during index hospitalization were excluded, given no examinable follow-up period. Written informed consent was obtained from all participants on admission. The institutional review board approved the study protocol.

### 2.2. TAVR Procedure

Patients with severe aortic stenosis with max velocity > 4.0 m/s, mean pressure gradient > 40 mmHg, or aortic valve area < 1.0 cm [2] were considered for TAVR following a multidisciplinary heart-valve team conference. The prosthesis type, size, and approach site were determined on the basis of pre-procedural echocardiographic and multi-detector computed tomographic findings. The type of anesthesia was determined according to the patients’ comorbidities.

All patients received TAVR according to the standard procedure. Patients received self-expandable valves (Corevalve, Evolut R, Evlolut PRO, or Evolut PRO+; Medtronic plc., Minneapolis, MN, USA) or balloon-expandable valves (Sapien XT or Sapien 3; Edwards Lifesciences Inc., Irvine, CA, USA) via trans-femoral, trans-aorta, trans-subclavian, or direct aorta approach under general or local anesthesia support. An antithrombotic regimen was used at the discretion of the clinicians.

### 2.3. Independent Variable and Primary Outcome

An independent variable was defined as the number of prescribed medication types at the index discharge. For example, if a patient received 2 tablets of carvedilol 2.5 mg per day and 3 tablets of enalapril 2.5 mg per day, the number of medications was counted as 2. According to previous analyses [11,12], we defined polypharmacy as medication number ≥10, which was assumed as an independent variable. Medications for hypertension, dyslipidemia, heart failure, coronary artery disease, stroke, peripheral artery disease, and atrial fibrillation were assumed as cardiovascular medications. Other medications were designated as non-cardiovascular medications. We further counted the number of potentially inappropriate medications raised in the updated Beers 2019 criteria [13].

Patients were followed for at most two years following the index discharge date, which we defined as day 0. Patients were followed at scheduled clinic visits at our institute or affiliated institutes by board-certified cardiologists. The primary outcome was mortality at two years. Secondary outcomes of interest were rate of heart failure readmission and all-cause readmission.

### 2.4. Clinical Variables

Demographics, laboratory, echocardiographic, and medication data obtained at the index discharge were designated as baseline characteristics. Standard laboratory data, including plasma B-type natriuretic peptide, were measured. Transthoracic echocardiography was performed routinely following TAVR to assess the implanted valve and overall cardiac function in a standard manner by expert sonographers, who were blinded to the daily clinical practice.

### 2.5. Statistical Analysis

Continuous variables were presented as median and interquartile range and compared using Mann-Whitney U test. Categorical variables were presented as numbers and percentages and compared using Fisher’s exact test. A value of 2-tailed *p* < 0.05 was considered statistically significant. Statistical analyses were performed using SPSS Statistics 22 (SPSS Inc., Armonk, IL, USA). The independent variable was polypharmacy, which was defined as medication number ≥10. The primary outcome was two-year all-cause death following the index discharge.

Cox proportional hazard ratio regression analyses were performed to investigate the impact of polypharmacy upon the primary outcome, which were adjusted for pre-specified potential confounders, including age, STS score, history of heart failure, estimated glomerular filtration rate, serum albumin, hemoglobin, plasma B-type natriuretic peptide, mini-mental state examination, and CSHA score. Cumulative incidences were compared between those with and without polypharmacy.

## 3. Results

### 3.1. Baseline Characteristics

A total of 352 patients were screened for inclusion. Of these, three patients who died during index hospitalization and four patients who were lost to follow-up following index discharge were excluded. The final study cohort consisted of 345 patients. Median age was 85 (83, 89) years old and 99 (29%) were men (Table 1). Median STS score was 4.6 (3.9, 6.1). Following TAVR, median peak velocity at aortic valve was 2.1 (1.7, 2.4) m/s and left ventricular ejection fraction was 64% (57%, 72%). Median plasma B-type natriuretic peptide level was 107 (57, 224) pg/mL. Mini-mental state examination was 26 (23, 28) points and CSHA score was 4 (3, 4) points.

### 3.2. Medication Number:

**Figure 1:**
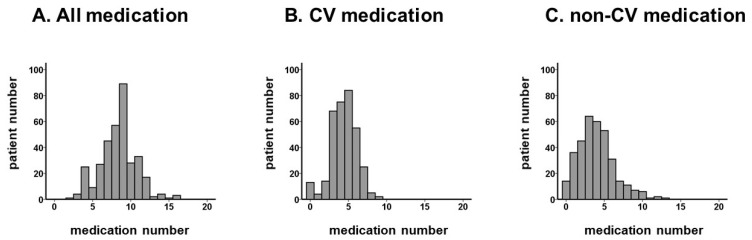
Distribution of medication numbers: all medication (**A**), CV medication (**B**), and non-CV medication (**C**). The number of medications was distributed widely. The distribution of CV medication is relatively narrower than for non-CV medication.CV, cardiovascular.

The numbers of prescribed medication types were distributed widely, with a median value of 9 (7, 10) (Figure 1A). Of the total, 31 patients (7%) received <5 of medications, 226 patients (54%) received medications ranging between 5 and 9, and 88 patients (21%) received ≥10 of medications, which was defined as polypharmacy.

The numbers of cardiovascular medications and non-cardiovascular medications are summarized in Figure 1B,C. The majority of patients were taking 4–7 cardiovascular medications.

**Figure 2:**
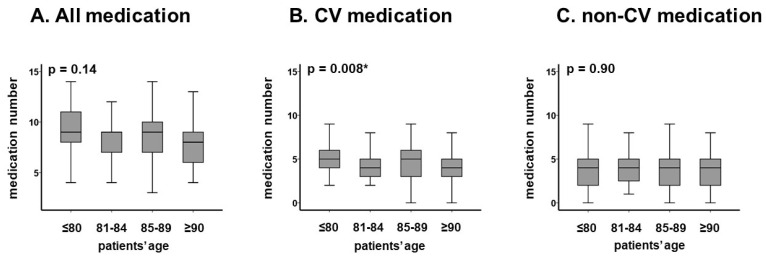
Distribution of median medication number in each age category: all medication (**A**), CV medication (**B**), and non-CV medication (**C**). The numbers of medications were not significantly stratified by age. The numbers of CV medications decreased as incremental age. The number of non-CV medications remained unchanged with incremental age. CV, cardiovascular. Median medication numbers were compared among each group using Wilcoxon signed-rank test. * *p* < 0.05.

The association between age and numbers of medications is displayed in Figure 2A–C. There was no significant association between age and the number of all medication (Figure 2A) and non-cardiovascular medication (Figure 2C) (*p* > 0.05 for both). The number of cardiovascular medications decreased as age increased (*p* = 0.008, Figure 2B).

### 3.3. Stratification of Patients’ Cohort by Polypharmacy

Patients were divided into two groups by the presence of polypharmacy (N = 88) (Table 1). There were no significant differences in demographic data, including age. The prevalence of major comorbidities was not significantly different between the two groups, except for the higher prevalence of coronary artery disease in patients with polypharmacy (36% versus 22%, *p* = 0.006). Patients with polypharmacy had lower serum albumin, worse renal function, lower cholesterol levels, and higher levels of plasma *B*-type natriuretic peptide levels compared with others (*p* < 0.05 for all). Frailty index, including mini-mental state examination and CSHA score, were not significantly different between those with and without polypharmacy. The number of potentially inappropriate medications tended to be higher in patients with polypharmacy compared with those without polypharmacy [3 (1, 4) versus 2 (1, 3); *p* = 0.087].

### 3.4. Impact of Polypharmacy on Clinical Outcomes

During the observational period (median 730 [356, 730] days), 21 patients died (4 from cardiovascular causes and 17 from non-cardiovascular causes; Table 2). The medication number was associated with 2-year mortality in unadjusted and adjusted models when it was assumed as a continuous variable or dichotomized variable (defined as presence or absence of polypharmacy, *p* < 0.05 for all; Table 3). Addition of one medication had 58% additional risk of mortality in the adjusted model (*p* < 0.001). The adjusted hazard ratio of polypharmacy for the 2-year mortality was 21.4 (95% confidence interval 6.06–74.8, *p* < 0.001).

**Figure 3:**
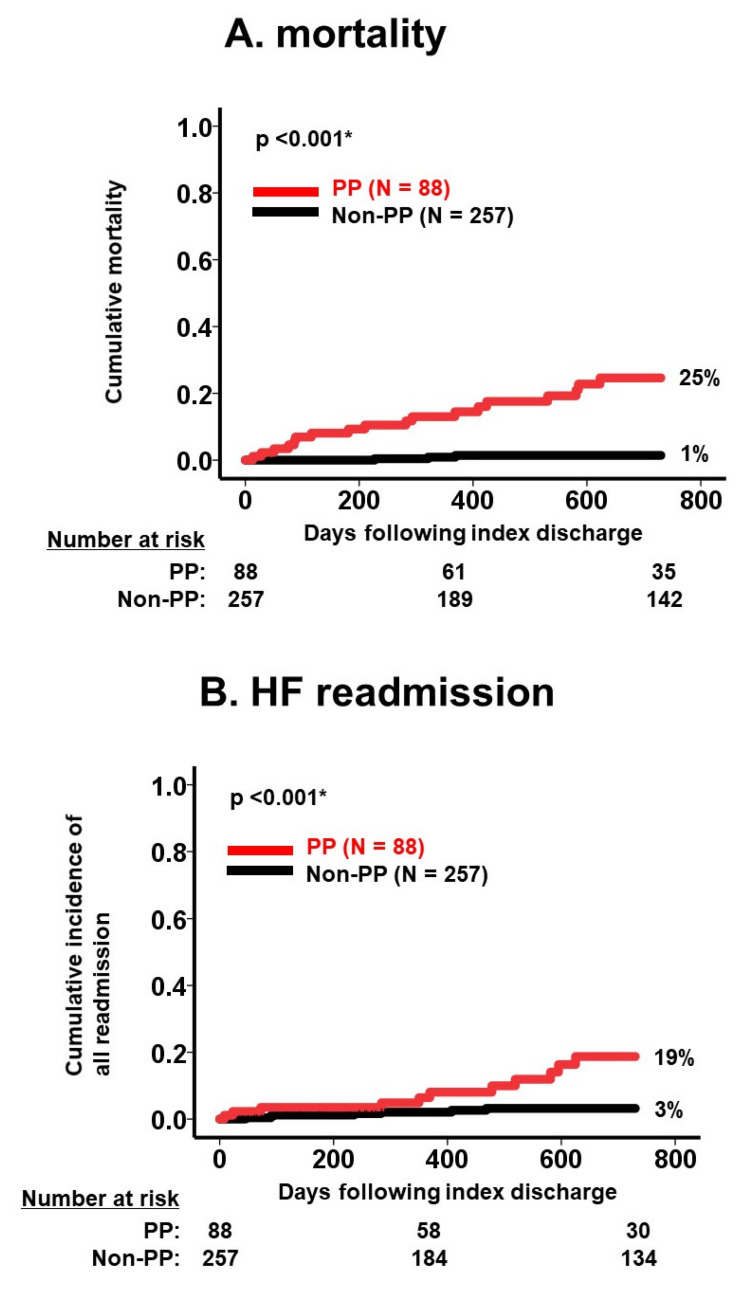

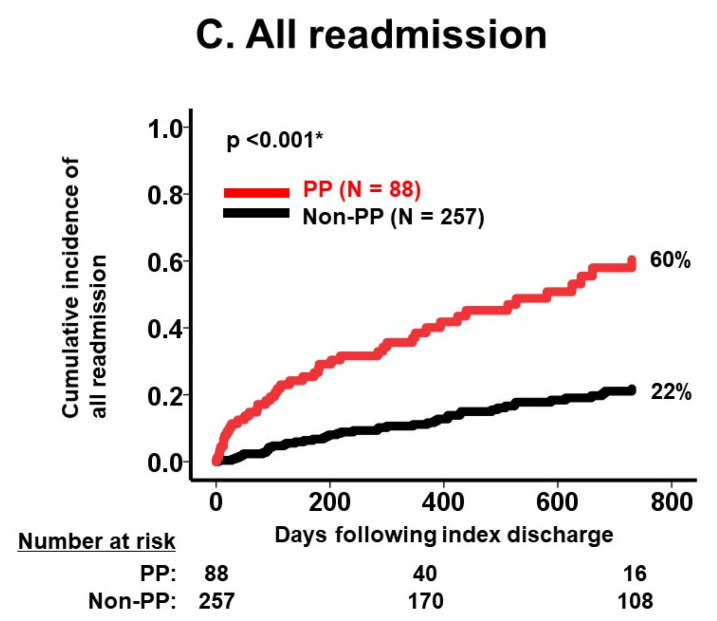
Cumulative incidence of death (**A**), HF readmission (**B**), and all readmission (**C**) stratified by PP versus non-PP. Cumulative incidences of each clinical outcome were significantly stratified by polypharmacy. HF, heart failure; PP, polypharmacy. Incidences were compared between the two groups using log-rank test. * *p* < 0.05.

During the same observational period, 18 patients experienced heart failure readmission. The medication number was similarly associated with a 2-year cumulative incidence of heart failure readmission (*p* < 0.05 for all; Table 3). There were 88 patients who were readmitted following index discharge due to a variety of reasons, including 11 malignancies, 10 fixtures, and 9 strokes (Table 2). Medication number was also associated with 2-year all-cause readmissions in the same manner (*p* < 0.05 for all; Table 2). The presence of polypharmacy was observed to stratify risk of 2-year mortality, the cumulative incidence of heart failure readmission, and the cumulative incidence of all-cause readmission (*p* < 0.05 for all; Figure 3A–C).

As a sub-analysis, the number of cardiovascular medications was not associated with 2-year mortality (hazard ratio 1.12, 95% confidence interval, 0.86–1.48, *p* = 0.46), whereas a higher number of non-cardiovascular medications was associated with an incremental increase in 2-year mortality, with a hazard ratio of 1.39 (95% confidence interval, 1.15–1.63, *p* < 0.001). The number of potentially inappropriate medications tended to be associated with 2-year mortality (hazard ratio 1.14, 95% confidence interval 0.82–1.32, *p* = 0.076).

## 4. Discussion

In this study, we investigated the prognostic impact of polypharmacy, which was defined as a medication number ≥10 at index discharge following TAVR, among an elderly cohort of patients with severe aortic stenosis. We observed a wide distribution of medications taken in the cohort. Polypharmacy was independently associated with an increased risk of 2-year mortality following TAVR. The number of cardiovascular medications was not associated with the primary outcome, whereas the number of non-cardiovascular medications had a significant association with the primary outcome.

### 4.1. Medication Number and Comorbidity

Given a correlation between increasing age and accumulation of comorbid conditions, the risk of polypharmacy and subsequent unintended harm to vulnerable cohorts is a commonly observed clinical dilemma [11,12]. The prevalence of polypharmacy varies depending on definition, accuracy of medication lists, and study population. In most studies of geriatric patient cohorts, a cutoff for medication numbers is set at ≥5, which traditionally defines polypharmacy [9,10]. Among patients with chronic heart failure where there are an expanding list of medications associated with mortality reduction, many patients often take ≥10 medications. In our cohort, 21% were taking ≥10 medications, consistent to what is seen in studies of patients with chronic heart failure [8]. This was the rationale for defining polypharmacy as medication number ≥10 in our study cohort.

Polypharmacy generally represents the existence of multiple comorbid conditions [6]. The presence of multiple major comorbid conditions was not significantly associated with polypharmacy, with the exception of coronary artery disease, which generally requires several essential medications including anti-platelets, beta-blockers, and statins for the secondary prevention. The majority of non-cardiovascular medications were prescribed for less severe comorbid conditions such as constipation and insomnia, whose prevalence is challenging in terms of accurate counting. Several frailty indices also were not significantly different between those with and without polypharmacy.

In comparison to other studies [14], medication numbers did not increase with increasing age. The number of cardiovascular medications, instead, was observed to decrease with increasing age, probably due to the age-related risk of drug-related complications such as worsening renal function.

### 4.2. Polypharmacy and Clinical Outcome

Polypharmacy was associated with morbidity and mortality in our cohort following TAVR. Thus, polypharmacy remains one of the unsolved issues, even following improvements in severe aortic stenosis. Causes of death were multifactorial. We did not identify heart failure as a common cause of death in this cohort. Health status can be improved significantly in patients following TAVR, but the residual risk of mortality and morbidity is strongly affected by baseline comorbid conditions, as demonstrated in prior studies of baseline frailty in patients receiving TAVR [4,15].

Polypharmacy was independently associated with worse clinical outcomes but still we cannot deny that it may represent the presence of underlying disease burden. Additionally, polypharmacy generally increases the risk of drug–drug and drug–disease interaction events, which can sometimes be unrecognized by clinicians given its complexity [6,16]. As examples of drug–drug interaction, multiple anti-hypertension agents might cause hypotension and falling. Multiple renin-angiotensin system inhibitors might progress chronic kidney disease and hyperkalemia. Duplicated anti-coagulation and anti-platelets might cause bleeding events. Fixture, renal failure and gastrointestinal bleeding were major causes of readmission in our cohort. Drug–disease interaction is another concern for this cohort. NSAIDs might worsen heart failure and chronic kidney disease. Benzodiazepine might increase the risk of falling and fixture in patients with high frailty. Non-dihydropyridine calcium channel blockers might worsen constipation. We should understand that clinical inertia by care teams may occur when it comes to de-prescribing therapies that other clinicians have prescribed for the patient.

### 4.3. Clinical Implication and Future Directions

Despite the innovation and significant clinical benefit of TAVR in patients who are deemed a higher surgical risk, many patients in this cohort have multiple baseline comorbid conditions, which may attenuate the intended survival benefit of the procedure. Given our findings, active attempts pre-discharge should be considered by care teams, including clinical pharmacists, in adjudicating medication lists and engaging with providers regarding the necessity of prescribed therapies and consideration of adverse interactions. By making this best-practice, it is possible that patients can experience a reduced medication burden, which in turn may minimize unintended downstream hazards and potentially increase the benefit of TAVR, although further studies are warranted to validate such strategies by conducting prospective interventional studies.

Interestingly, the number of cardiovascular medications did not have a negative prognostic impact. Although we should care about drug-related adverse events such as hyperkalemia, particularly in elderly patients with chronic kidney disease [16], appropriate up-titration of cardiovascular medication might not necessarily need to be hesitated in this cohort [17]. Again, safety and efficacy of such an aggressive up-titration of cardiovascular medication requires further prospective studies to be validated.

On the contrary, we should attempt to minimize the number of non-cardiovascular medications. Particularly, potentially inappropriate medication would be the target of intervention. Polypharmacy is associated with “doctor shopping”. A definite hospital–clinic relationship should be established. A screening tool to adjust medication is highly encouraged to minimize medication numbers [18]. Educative activity to encourage clinicians to prescribe dietary and exercise therapy prior to medication would be effective. Aggressive intervention by pharmacists to survey medication lists would also be practical. The clinical implication of aggressive minimization of medication numbers among those receiving TAVR remains the next concern.

### 4.4. Limitations

This is a retrospective study consisting of a moderate sample size. We performed multivariate analyses but other unadjusted potential confounders may also have impacted risk for the primary endpoint. We considered restricted numbers of variables for the adjustment, given the small sample size. We collected data on major comorbid conditions such as hypertension, but did not assess for more minor conditions such as constipation, which might also have had considerable impact on the findings. Given the retrospective nature of this analysis, understanding the true association between medication number and comorbidities remains uncertain. We focused on the number of medications, and did not determine any specific medication that may have had a negative prognostic impact. We also did not account for the doses and numbers of tablet. We only focused on the number of medication types. The presence of over-the-counter medications was not assessed in this analyses. Given the multiple causes of death in this analysis, we could not assess detailed causality between medication numbers and each cause of death.

## 5. Conclusions

Polypharmacy was independently associated with worse morbidity and mortality following TAVR among elderly patients with multi-comorbidities, including severe aortic stenosis. On the contrary, given that the number of cardiovascular medications did not have negative prognostic impact, it might not be necessary to hesitate to administer cardiovascular medications in this cohort. The clinical implications of interventions designed to decrease medication numbers in this cohort (all types of medication or non-cardiovascular medication) needs to be prospectively studied.

## Figures and Tables

**Table 1:** Baseline characteristics at index discharge.

	Total (N = 345)	PP (N = 88)	Non-PP (N = 257)	*p* Value
Demographics				
Age, years	85 (83, 89)	85 (81, 88)	85 (83, 88)	0.27
Men	99 (29%)	27 (31%)	72 (28%)	0.36
Body surface area, m^2^	1.38 (1.28, 1.50)	1.37 (1.25, 1.51)	1.39 (1.29, 1.50)	0.43
STS score	4.6 (3.9, 6.1)	4.7 (4.0, 6.3)	4.6 (3.9, 6.0)	0.34
Vital signs				
Systolic blood pressure, mmHg	117 (106, 128)	117 (102, 125)	117 (106, 128)	0.32
Pulse rate, bpm	70 (63, 78)	68 (63, 77)	70 (63, 78)	0.83
Comorbidity				
Atrial fibrillation	44 (13%)	11 (13%)	33 (13%)	0.55
Hypertension	252 (73%)	64 (73%)	188 (73%)	0.52
Diabetes mellitus	61 (18%)	18 (20%)	43 (17%)	0.26
Dyslipidemia	165 (48%)	46 (52%)	119 (46%)	0.20
Coronary artery disease	88 (26%)	32 (36%)	56 (22%)	0.006 *
History of stroke	45 (13%)	13 (15%)	32 (12%)	0.35
History of heart failure	138 (40%)	40 (45%)	98 (38%)	0.14
Chronic obstructive pulmonary disease	21 (6%)	6 (7%)	15 (6%)	0.46
Peripheral artery disease	77 (22%)	25 (28%)	52 (20%)	0.076
Frailty				
Mini-mental state examination, points	26 (23, 28)	26 (23, 28)	26 (24, 29)	0.38
CSHA score, points	4 (3, 4)	4 (3, 4)	4 (3, 5)	0.52
Echocardiography				
Aortic valve peak velocity, m/s	2.1 (1.7, 2.4)	2.1 (1.8, 2.4)	2.0 (1.7, 2.4)	0.62
LVDd, mm	45 (41, 49)	46 (42, 49)	45 (41, 49)	0.45
Left ventricular ejection fraction, %	64 (57, 72)	64 (59, 72)	64 (57, 71)	0.45
Laboratory data				
Hemoglobin, g/dL	10.4 (9.7, 11.1)	10.4 (9.6, 11.1)	10.4 (9.7, 11.3)	0.59
Serum albumin, g/dL	3.4 (3.0, 3.6)	3.3 (2.9, 3.5)	3.4 (3.1, 3.7)	0.013 *
Serum sodium, mEq/L	139 (138, 141)	140 (138, 141)	139 (137, 141)	0.39
Serum potassium, mEq/L	4.3 (4.0, 4.6)	4.3 (4.0, 4.4)	4.3 (4.0, 4.6)	0.32
eGFR, mL/min/1.73 m^2^	49.4 (35.7, 62.4)	44 (32, 57)	52 (38, 66)	0.006 *
Total cholesterol, mg/dL	157 (135, 173)	148 (131, 165)	158 (137, 174)	0.026 *
Low density lipoprotein cholesterol, mg/dL	88 (2, 105)	83 (66, 95)	89 (73, 109)	0.024 *
Plasma B-type natriuretic peptide, pg/mL	107 (57, 224)	118 (68, 294)	94 (55, 196)	0.041 *
C-reactive protein, mg/dL	0.7 (0.3, 2.0)	1.2 (0.4, 2.8)	0.6 (0.3, 1.9)	0.36

PP, polypharmacy; STS, society of thoracic surgeons; CSHA, Canadian study of health and aging; LVDd, left ventricular end-diastolic diameter; eGFR, estimated glomerular filtration rate. Continuous variables are stated as median and interquartile and compared between the two groups using Mann-Whitney U test. Categorical variables are stated as number and percentage and compared between the two groups using Fisher’s exact test. * *p* <0.05.

**Table 2:** Causes of death or readmission.

	Death (N = 21)	Readmission (N = 88)
Cardiovascular		
Heart failure	2	18
Stroke	1	9
Sudden death	1	0
Arrhythmia	0	5
Non cardiovascular		
Unknown or others	7	5
Infection	7	22
Fixture	0	10
Renal failure	3	3
Malignancy	0	11
Gastrointestinal bleeding	0	5

**Table 3:** Impact of polypharmacy on 2-year endpoints.

	Unadjusted Analyses		Adjusted Analyses	
	Hazard Ratio (95% CI)	*p* Value	Hazard Ratio (95% CI)	*p* Value
Mortality				
Medication number per one medicine	1.51 (1.24–1.79)	<0.001 *	1.58 (1.24–1.88)	<0.001 *
PP versus non-PP	19.5 (5.61–65.6)	<0.001 *	21.4 (6.06–74.8)	<0.001 *
HF readmission				
Medication number per one medicine	1.31 (1.06–1.58)	0.005 *	1.29 (1.03–1.58)	0.018 *
PP versus non-PP	5.21 (2.01–13.9)	0.001 *	4.52 (1.63–12.9)	0.004 *
All readmission				
Medication number per one medicine	1.24 (1.13–1.45)	<0.001 *	1.29 (1.15–1.34)	<0.001 *
PP versus non-PP	3.76 (2.56–5.89)	<0.001 *	3.39 (2.12–5.39)	<0.001 *

PP, polypharmacy; HF, heart failure; CI, confidence interval. Medication number was dealt with as a continuous variable and categorical variable (i.e., polypharmacy defined as medication numbers ≥10 versus non-polypharmacy). Analyses were adjusted for potential confounders including age, STS score, history of heart failure, estimated glomerular filtration rate, serum albumin, hemoglobin, plasma B-type natriuretic peptide, MMSE, and CSHA scale. * *p* < 0.05.

## Data Availability

Data are available from the corresponding author upon reasonable request.
